# Effects of Nitrate on Hydrogenogenic Carbon Monoxide Oxidation in *Parageobacillus thermoglucosidasius*

**DOI:** 10.1111/1758-2229.70133

**Published:** 2025-06-13

**Authors:** Yuka Adachi Katayama, Yoshinari Imaura, Masao Inoue, Shunsuke Okamoto, Yoshihiko Sako, Ryoma Kamikawa, Takashi Yoshida

**Affiliations:** ^1^ Graduate School of Agriculture Kyoto University Kyoto Japan; ^2^ R‐GIRO Ritsumeikan University Kusatsu Shiga Japan; ^3^ College of Life Sciences Ritsumeikan University Kusatsu Shiga Japan

**Keywords:** bioenergetics, carbon monoxide dehydrogenase, *Parageobacillus*

## Abstract

*Parageobacillus thermoglucosidasius* is a thermophilic facultative anaerobe capable of hydrogenogenic carbon monoxide (CO) oxidation utilising nickel‐containing CO dehydrogenase (Ni‐CODH) and energy‐converting hydrogenase (ECH). Nitrates have been reported to exert promoting or inhibitory effects on the growth of CO oxidizers and acetogens, and these contradictory outcomes obscure the relationship between nitrate and CO oxidation. In this study, we analysed the effects of nitrate on hydrogenogenic CO oxidation and growth in *P. thermoglucosidasius* NBRC 107763^T^ using wild‐type and *codh*‐ and/or *ech‐*disrupted strains. The results demonstrated that the addition of 50 mM nitrate suppressed hydrogenogenic CO oxidation while promoting hydrogen‐oxidising nitrate reduction and rapid cell growth, resulting in a 2.3‐fold higher OD_600_ than the control. Assays using cell lysates showed that 10 μM nitrate suppressed CO oxidation below the detection limit without affecting hydrogen production, indicating that nitrate affects the CO‐oxidising function. These findings imply that CO oxidation in *P*. *thermoglucosidasius* is primarily coupled to proton reduction, and deactivated during nitrate respiration. Therefore, hydrogenogenic CO oxidation serves as an auxiliary energy‐obtaining mechanism, functioning in the absence of alternative electron acceptors such as nitrate. This study enhances our understanding of CO‐dependent energy generation and highlights the supplemental use of CO in *P*. *thermoglucosidasius*.

## Graphical abstract

*Parageobacillus thermoglucosidasius* hierarchically utilises nitrate respiration and hydrogenogenic carbon monoxide (CO) oxidation. Nitrate suppresses CO oxidation and promotes rapid cell growth through hydrogen‐dependent nitrate reduction. For this bacterium, CO serves as a supplemental energy source in environments lacking alternative electron acceptors such as nitrate.

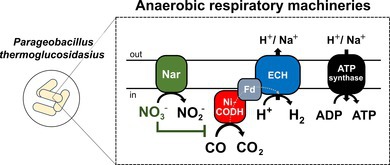

## 1 Introduction

The genus *Parageobacillus* (formerly *Geobacillus*) is a group of thermophilic facultative anaerobes belonging to the family *Bacillaceae*. Members of this genus grow at temperatures ranging from 37°C to 75°C, with optimal temperatures of 55°C to 65°C (Najar and Thakur 2020). Because of their ability to form spores (Najar and Thakur 2020) and survive at low temperatures (Cockell et al. 2015; Milojevic et al. 2022), *Parageobacillus/Geobacillus* species have been found in diverse regions and environments (Portillo et al. 2012; Milojevic et al. 2022). The type species of this genus, *P*. *thermoglucosidasius*, has been isolated from a wide range of regions, including cold and hot environments (e.g., sea sediments, soils, and hot springs) across all continents except Antarctica (Suzuki et al. 1983; Brumm et al. 2015; Inoue, Tanimura, et al. 2019). In addition to its broad distribution, *P. thermoglucosidasius* has gained attention because of its utility as a model organism for metabolic engineering, particularly for bioethanol production and lignocellulose degradation (Taylor et al. 2009; Paredes‐Barrada et al. 2024). Moreover, this bacterium is the first thermophilic facultative anaerobe that produces hydrogen (H_2_) in a coupled reaction with carbon monoxide (CO) oxidation, a reaction known as hydrogenogenic CO oxidation or water‐gas shift reaction (CO + H_2_O ⇔ CO_2_ + H_2_) (Mohr, Aliyu, Küchlin, Polliack, et al. 2018). Two strains, TG4 and B1‐2, have been isolated by cultivation under gas environments containing CO (Inoue, Tanimura, et al. 2019; Nishida et al. 2024). Since H_2_ is a promising source of renewable energy, efforts to produce H_2_ from CO‐rich waste gases or synthesis gases using *P. thermoglucosidasius* for industrial applications have gained research attention (Mohr, Aliyu, et al. 2019; Mohr, Infantes, et al. 2019; Adachi et al. 2020; Mol et al. 2024).

Hydrogenogenic CO oxidation is an energy‐conserving process catalysed by nickel‐containing CO dehydrogenase (Ni‐CODH) and energy‐converting hydrogenase (ECH) (Fukuyama et al. 2020). Ni‐CODH, which is classified into clade A to H based on their structure and phylogeny (Inoue, Nakamoto, et al. 2019; Inoue et al. 2022), catalyses the oxidation of CO to CO_2_ (𝐸^0′^ = −520 mV, CO_2_/CO), while ECH catalyses the reduction of protons to H_2_ (𝐸^0′^ = −410 mV, H^+^/H_2_) (Schuchmann and Müller 2014). In the process of hydrogenogenic CO oxidation, ferredoxin (Fd)‐like protein (CooF) mediates the transfer of electrons generated from CO oxidation by Ni‐CODH to ECH, which reduces protons and drives the translocation of Na^+^/H^+^ across the cell membrane, generating a chemiosmotic ion gradient that can subsequently be used for ATP synthesis (Schoelmerich and Müller 2019). Since the enzymes can consume CO at nearly the diffusion‐limited rate, and many sulfate reducers containing Ni‐CODH/ECH are sensitive to CO, hydrogenogenic CO oxidation has been believed to play a role in CO detoxification (Ragsdale 2004; Oelgeschläger and Rother 2008; Parshina et al. 2010; Robb and Techtmann 2018; Fukuyama et al. 2020). In the genome of *P. thermoglucosidasius*, genes for Ni‐CODH, CooF, and ECH form a gene cluster (Mohr, Aliyu, Küchlin, Polliack, et al. 2018), while the absence of CO‐sensing transcriptional factors indicates CO‐independent regulation of Ni‐CODH/ECH in this species (Mohr, Aliyu, Küchlin, Polliack, et al. 2018; Mohr, Aliyu, Küchlin, Zwick, et al. 2018). In a previous study, these genes in *P. thermoglucosidasius* were disrupted to establish the Δ*codh*, Δ*ech*, and Δ*codh*–*ech* strains, demonstrating that both Ni‐CODH and ECH are essential for growth via hydrogenogenic CO oxidation (Adachi et al. 2020). However, the physiological role of Ni‐CODH/ECH in this facultative anaerobe remains poorly understood.

In many hydrogenogenic CO oxidizers, the low redox potential of CO drives not only proton reduction but also the reduction of other electron acceptors (Fukuyama et al. 2020). For instance, CO‐derived electrons are transferred to sulphate, thiosulfate, and metal ions possibly via electron transport chains (Oelgeschläger and Rother 2008; Fukuyama et al. 2018; Robb and Techtmann 2018), or indirectly via H_2_, which reduces terminal electron acceptors such as sulphate and fumarate (Oelgeschläger and Rother 2008; Eckert et al. 2019). Among the electron acceptors, nitrate is utilised by many microbes in anoxic environments such as soils and marine sediments (Kuypers et al. 2018). Indeed, nitrate‐reducing ability has been observed in many CO oxidizers, including *Moorella thermoacetica* , *Carboxydothermus hydrogenoformans* , and *P. thermoglucosidasius* (Suzuki et al. 1983; Seifritz et al. 2003; Henstra and Stams 2004). A previous study showed that *Parageobacillus* sp. G301, which contains oxygen‐tolerant molybdenum‐containing CODH (Mo‐CODH) in addition to Ni‐CODH, couples CO oxidation with the reduction of nitrate to nitrite (𝐸^0′^ = +430 mV, NO_3_^−^/NO_2_^−^) (Imaura et al. 2023). While Mo‐CODH is typically involved in CO‐oxidising nitrate reduction (Imaura et al. 2023), Ni‐CODH has also been reported to be involved in the reaction (Slobodkin et al. 2019). However, the relationship between Ni‐CODH and nitrate has been a topic of debate. For instance, CO oxidation is coupled to the reduction of nitrate to ammonium in *Deferribacter autotrophicus* (Slobodkin et al. 2019). However, nitrate inhibits both hydrogenogenic CO oxidation and CO‐dependent growth in *Carboxydocella thermautotrophica* strain 019 (Toshchakov et al. 2018). The effect of nitrate on acetogens, which harbour Ni‐CODHs as a key enzyme in the autotrophic Wood–Ljungdahl pathway (WLP), has also been shown to vary: nitrate enhanced CO‐dependent growth using WLP in *Clostridium ljungdahlii* , had no impact in *Thermoanaerobacter kivui* , and inhibited it in *M. thermoacetica* (Fröstl et al. 1996; Drake and Daniel 2004; Emerson et al. 2019). The reasons for these conflicting results remain unclear.

Despite reports showing that nitrate can influence CO utilisation in some bacteria containing Ni‐CODH, the exact nature of this relationship remains unclear, partially because of the lack of genetic engineering‐based analyses. *P. thermoglucosidasius* possesses genes for nitrate reductase (Nar; AOT13_11770) and nitrite reductase (Nir; AOT13_07200) (Brumm et al. 2015), and nitrate reduction has been previously confirmed in this bacterium (Suzuki et al. 1983). Thus, coupling CO oxidation and nitrate/nitrite reduction could be an option for efficient energy conservation for this bacterium. Therefore, we aimed to elucidate the effects of nitrate on the growth and CO oxidation activity of *P. thermoglucosidasius* using *codh*‐ and *ech*‐deleted strains to enhance our understanding of the role of CO‐dependent respiration in this bacterium.

## 2 Materials and Methods

### 2.1 Organisms

*P. thermoglucosidasius* NBRC 107763^T^ (=DSM 2542^T^) wild‐type strain (Suzuki et al. 1983) was purchased from the Biological Resource Center of the National Institute of Technology and Evaluation (NBRC) (Japan). The Δ*codh*, Δ*ech*, and Δ*codh*–*ech* strains were established using the wild‐type strain and maintained in the laboratory as described previously (Adachi et al. 2020).

### 2.2 Culture Experiments

Culture experiments were performed using a basal medium (Adachi et al. 2020), containing 0.3 g KCl (28538‐62, Nacalai Tesque Inc., Kyoto, Japan), 0.5 g NH_4_Cl (02424‐55, Nacalai Tesque), 0.1 g KH_2_PO_4_ (2872155, Nacalai Tesque), 0.2 g MgCl_2_･6H_2_O (2090865, Nacalai Tesque), 0.1 g CaCl_2_･2H_2_O (0673015, Nacalai Tesque), 0.03 g sodium silicate (190‐03215, Wako Pure Chemical Industrials, Kyoto, Japan), 0.1 g NaHCO_3_ (191‐01305, Nacalai Tesque), 0.5 mL trace element solution SL6 (Pfennig 1974), 1 mL vitamin solution (Wolin et al. 1963) per litre, with the concentration of yeast extract (212750, BD Biosciences, CA, USA) modified to 0.01%. The wild‐type strain was cultivated at 65°C and 100 rpm in glass bottles sealed with bromobutyl rubber stoppers and phenol resin screw caps, with each bottle containing 100 mL of the medium and 200 mL of a gas mixture composed of 25% CO and 75% N_2_ (both from Kindgas, Kyoto, Japan). KCl (control; 2851475, Nacalai Tesque), KNO_3_ (2870485, Nacalai Tesque), or KNO_2_ (2873585, Nacalai Tesque) was added to the medium at final concentrations of 10–50 mM. Additionally, 50 mM sodium pyruvate (2980625, Nacalai Tesque) was added to the culture medium as an electron donor and carbon source, along with 25 mM MOPS‐NaOH buffer (pH 6.8, 65°C) (2341525, Nacalai Tesque), which mitigates pH decreases by fermentation products, when mentioned. Cell growth was monitored by measuring the optical density at 600 nm (OD_600_) using an Ultrospec 2100 Pro (Biochrom, Berlin, Germany). The doubling time was calculated from the cell growth curve during the log phase, specifically from 5 to 19 h for first growth and from 25.5 to 30 h for second growth.

The headspace gas composition was analysed using a GC‐2014 gas chromatography system (Shimadzu, Kyoto, Japan) equipped with a thermal conductivity detector and a Shincarbon ST packed column (2.0 m × 3.0 mm, Shinwa Chemical Industries, Kyoto, Japan), using argon as carrier gas (Kindgas) with a column temperature of 40°C–200°C. The amounts of CO, H_2_, and CO_2_ were calculated using standard curves generated from measurements of gas mixtures containing 0.5%, 1%, 5%, 25%, 50%, and 100% of high purity CO or H_2_, and 1%, 5%, 10%, and 20% of high purity CO_2_ or O_2_ (all of them with 99.9% purity from Kindgas), with triplicate samples for each concentration. The dissolved concentrations of gases in the liquid phase were calculated according to Henry's law and van't Hoff equation (65°C, 1 atm), using estimated Henry's constants of 1.4 × 10^−5^ for CO, 9.2 × 10^−6^ for H_2_, 6.9 × 10^−4^ for CO_2_, and 2.1 × 10^−5^ for O_2_ (Sander 2023). For cultures with nitrate, the concentrations of nitrate and nitrite in the liquid phase were quantified by the Griess reaction using the NO_2_/NO_3_ Assay Kit CII (344‐07991, Dojindo Laboratories, Kumamoto, Japan). Statistical analysis was performed using the unpaired Welch's *t‐*test, and multiplicity was adjusted using the Holm–Bonferroni method in Microsoft Excel 2021.

### 2.3 CO Oxidation and H_2_ Production Assays With Whole‐Cell Lysates

CO oxidation and H_2_ production were measured using whole‐cell lysates. The wild‐type, Δ*codh*, Δ*ech*, and Δ*codh*–*ech* strains were grown in 100 mL of liquid TGP medium (Cripps et al. 2009) under 25% CO and 75% air at 65°C, 100 rpm (*n* = 3). TGP medium contains 17 g tryptone (3564095, Nacalai Tesque), 3 g soypeptone (2644365, Nacalai Tesque), 4 mL glycerol (1701735, Nacalai Tesque), 4 g sodium pyruvate, 5 g NaCl (3132005, Nacalai Tesque), and 2.5 g K_2_HPO_4_ (2872155, Nacalai Tesque) per litre. The cultures were incubated in a shaker at 100 rpm to allow the cells to express Ni‐CODH/ECH if the genes were present (Mohr, Aliyu, Küchlin, Polliack, et al. 2018). After 24 and 45 h of cultivation, the headspace gas composition of the bottles containing the wild‐type strain was analysed using gas chromatography to verify hydrogenogenic CO oxidation. Cells from 50 mL of the culture were collected by centrifugation at 7142× *g* in an anaerobic chamber (Coy Laboratory, MI, USA). The cell pellet was then suspended in 50 mL of a buffer containing 100 mM potassium phosphate (20°C, pH 6.8), 2 mM ethylenediaminetetraacetic acid (EDTA), and 1 μM sodium hydrosulfite (HS) (all of them from Nacalai Tesque), and lysed by five rounds of ultrasonication at 20 W for 30 s at room temperature.

For measuring CO oxidation in the lysates, methyl viologen (MV_ox_) (𝐸^0′^ = −450 mV, MV_ox_/MV_red_) (2324661, Nacalai Tesque) was used as an electron acceptor, as previously described (Bonam et al. 1989; Kerby et al. 1992; Singer et al. 2006; Ainala et al. 2017; Gencic and Grahame 2020; Song et al. 2020). A final concentration of 6.9 mM of MV_ox_ was added to 5 mL of the whole‐cell lysate in a 13.5‐mL serum bottle sealed with a rubber stopper and a polypropylene screw cap and incubated under 5.6% CO and 94.4% N_2_ at 65°C for 24 h.

To measure H_2_ production in the lysates, MV_red_ was used as an electron donor, as described in previous studies (Kerby et al. 1992; Singer et al. 2006; Ainala et al. 2017). In this assay, 1.4 mM MV and 56 mM HS were added to 5 mL of the lysates in a 13.5‐mL serum bottle sealed with a rubber stopper and a polypropylene screw cap, and incubated under 100% N_2_ at 65°C for 5 h. KCl or KNO_3_ was added at the final concentration of 10 μM when mentioned. The headspace concentrations of CO and H_2_ at the beginning and end of the reaction were measured by gas chromatography. The protein content in the whole‐cell lysate was measured by the Bradford method (Bradford 1976) using a Bio‐Rad Protein Assay (Bio‐Rad, CA, USA) with bovine serum albumin as the standard. The CO consumption was calculated by subtracting the final CO amount from the initial amount. Comparisons between the wild‐type and genetically engineered strains were performed using the Tukey's test in Microsoft Excel 2021.

### 2.4 Classification of Ni‐CODH

Ni‐CODH‐containing species with clear reports of nitrate effects are listed on the basis of previous literature (Table 1). Species showing clear indications of nitrate effects on CO consumption and/or WLP utilisation were included, whereas those with only data on nitrate reduction in the presence of CO were excluded. All 39 CODHs from the 13 species were classified into clades A to H and mini‐CODH, as described previously (Inoue, Nakamoto, et al. 2019; Katayama et al. 2024) and based on the previous comprehensive survey by Inoue, Nakamoto, et al. (2019). Briefly, full‐length sequences of Ni‐CODH (CooS/CdhA) were aligned using mafft v7.525 (Katoh and Standley 2013) with the E‐INS‐I application and trimmed using trimAI v1.4.1 (Capella‐Gutiérrez et al. 2009). A phylogenetic tree was constructed using IQ‐TREE v2.3.6 (Minh et al. 2020), which was then visualised in MEGA7 (Kumar et al. 2016).

**TABLE 1:** Summary of the effects of nitrate on species using Ni‐CODH.

Phyla/classes/orders	Organisms	Ni‐CODH IDs	Ni‐CODH clades	Function of the neighbouring genes	Nitrate reductases (NO_3_^−^ → end products)	References
Species with nitrate‐suppressive effect on acetogenesis or hydrogenogenic CO oxidation						
Bacillota/bacilli/bacillales	*Parageobacillus thermoglucosidasius*	WP_013400775	F	ECH	Nar (NO_3_^−^ → NO_2_^−^)	This study
Bacillota/clostridia/thermoanaerobacterales	*Carboxydothermus pertinax*	WP_075859348	F	ECH	—^b^	(Yoneda et al. 2012; Fukuyama et al. 2018)
		WP_075858965	F	WLP^a^		
		WP_075858402	F	—		
		WP_075859600	D	—		
Bacillota/clostridia/eubacteriales	*Carboxydocella thermautotrophica*	WP_078665807	F	WLP	—^b^	(Fröstl et al. 1996; Toshchakov et al. 2018)
		WP_078665961	F	—		
		WP_078665971	F	—		
		WP_078666160	F	—		
		WP_078664291	D	—		
		WP_078664535	D	—		
Bacillota/clostridia/natranaerobiales	*Natranaerofaba carboxydovora*	WP_241079318	F	WLP	Nap (NO_3_^−^ → NH_4_^+^)	(Sorokin et al. 2021)
		WP_241080517	F	—		
		WP_241079570	D	—		
Bacillota/clostridia/moorellales	*Moorella thermoacetica*	WP_053104382	F	WLP	Nar (NO_3_^−^ → NH_4_^+^)	(Fröstl et al. 1996)
		WP_162490292	B	—		
Species coupling CO oxidation with nitrate reduction						
Deferribacterota/deferribacteres/deferribacterales	*Deferribacter autotrophicus*	WP_149267424	E	—	Nap (NO_3_^−^ → NH_4_^+^)	(Slobodkin et al. 2019)
		WP_149267451	E	—		
Species capable of acetogenic growth in the presence of nitrate						
Bacillota/clostridia/thermoanaerobacterales	*Thermoanaerobacter kivui*	WP_049685786	E	WLP	—	(Fröstl et al. 1996)
		WP_049684818	C	—		
Bacillota/clostridia/eubacteriales	*Acetobacterium woodii*	WP_014355463	E	WLP	—	(Fröstl et al. 1996)
		WP_014356284	C	—		
Bacillota/clostridia/eubacteriales	*Clostridium aceticum*	WP_044823348	E	WLP	—	(Fröstl et al. 1996)
		WP_044823439	E	—		
		WP_044824692	E	—		
		WP_044823808	C	—		
Bacillota/clostridia/eubacteriales	*Clostridium ljungdahlii*	WP_013240371	E	WLP	? (NO_3_^−^ → NH_4_^+^)	(Emerson et al. 2019)
		WP_013238446	D	—		
		WP_013237576	C	—		
Bacillota/clostridia/eubacteriales	*Clostridium formicaceticum*	WP_081561958	E	WLP	—	(Fröstl et al. 1996)
		WP_070967604	E	—		
		WP_070964405	E	—		
		WP_070969114	C	—		
		WP_070971793	C	—		
		WP_070966689	D	—		
Bacillota/clostridia/lachnospirales	*Blautia producta*	WP_018597549	E	WLP	—	(Fröstl et al. 1996)
		WP_018598141	B	—		
		WP_018597958	C	—		
Euryarchaeota/archaeoglobi/archaeoglobales	*Ferroglobus placidus*	WP_012965244	A	WLP	Nar (NO_3_^−^ → NO_2_^−^, NO)	(Vorholt et al. 1997)

^a^ Autotrophic growth using WLP was not confirmed in *C. pertinax* , in which genome the Ni‐CODH/ACS‐encoding gene contains a frameshift mutation, while hydrogenogenic CO oxidation was confirmed.

^b^ Nitrate reduction using electron donors such as hydrogen and organic substrates was not tested.

## 3 Results

### 3.1 Hydrogenogenic CO Oxidation Was Suppressed by Addition of Nitrate

**FIGURE 1:**
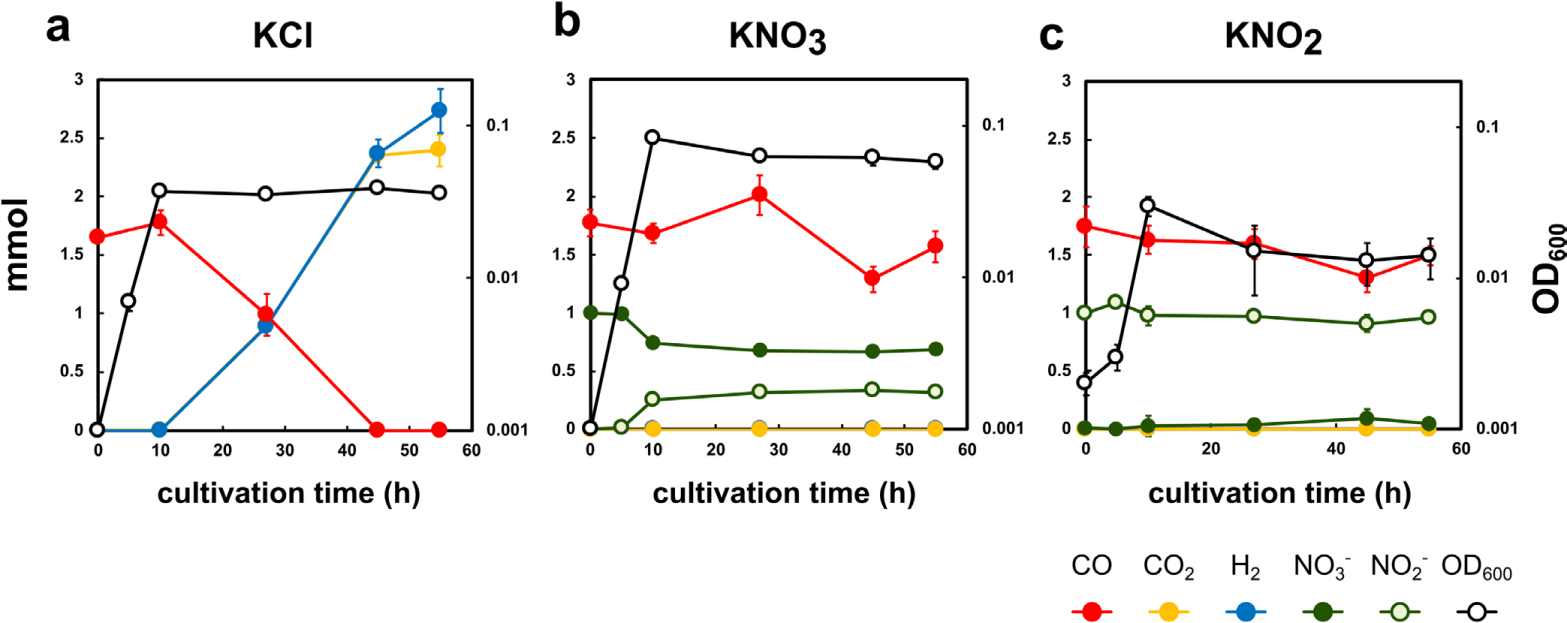
Growth of the *P. thermoglucosidasius* wild‐type strain under CO and electron acceptors. The wild‐type strain was cultivated under 25% CO and 75% N_2_ atmosphere in a basal medium supplemented with 0.01% yeast extract. Either 10 mM KCl (a), KNO_3_ (b), or KNO_2_ (c) was added to the medium. The figures show the total amounts of CO (red), CO_2_ (yellow), H_2_ (blue), NO_3_^−^ (green), and NO_2_^−^ (light green with green lines) and the OD_600_ values (black lines). The experiment was performed in triplicate. The error bars represent the standard error of the mean.

The wild‐type strain of this bacterium was first cultured in basal medium supplemented with 1.0 mmol (10 mM) of KCl, KNO_3_, or KNO_2_ under a 25% CO and 75% N_2_ headspace atmosphere in a medium containing 0.01% yeast extract (*n* = 3). In this setup, CO serves as an electron donor, NO_3_^−^/NO_2_^−^ potentially serves as electron acceptors, and yeast extract potentially provides nutrients including carbon and additional electrons. In the KCl control, 1.66 ± 0.04 mmol CO was consumed and 2.59 ± 0.19 mmol H_2_ and 2.40 ± 0.13 mmol CO_2_ were produced at 55 h post‐cultivation (mean ± standard error) (Figure 1a). In contrast, no apparent consumption of CO and no production of H_2_ or CO_2_ were observed in both KNO_3_‐containing and KNO_2_‐containing cultures (Figure 1b,c), indicating that hydrogenogenic CO oxidation was not activated under these conditions. Cell growth was observed until 10 h, with OD_600_ values of 0.038 ± 0.001, 0.082 ± 0.002, and 0.033 ± 0.004 in the presence of KCl, KNO_3_, and KNO_2_, respectively (Figure 1a–c). In the medium containing KNO_3_, 0.31 ± 0.04 mmol NO_3_^−^ was reduced to 0.31 ± 0.04 mmol NO_2_^−^ (Figure 1b), suggesting that the enhanced growth under this condition was attributable to nitrate respiration, which could be coupled to electron donation from either the yeast extract or a non‐detectable level of CO. No apparent NO_2_^−^ consumption was detected in the medium containing KNO_2_ (Figure 1c), suggesting that NO_2_^−^ did not serve as an electron acceptor in this condition.

**FIGURE 2:**
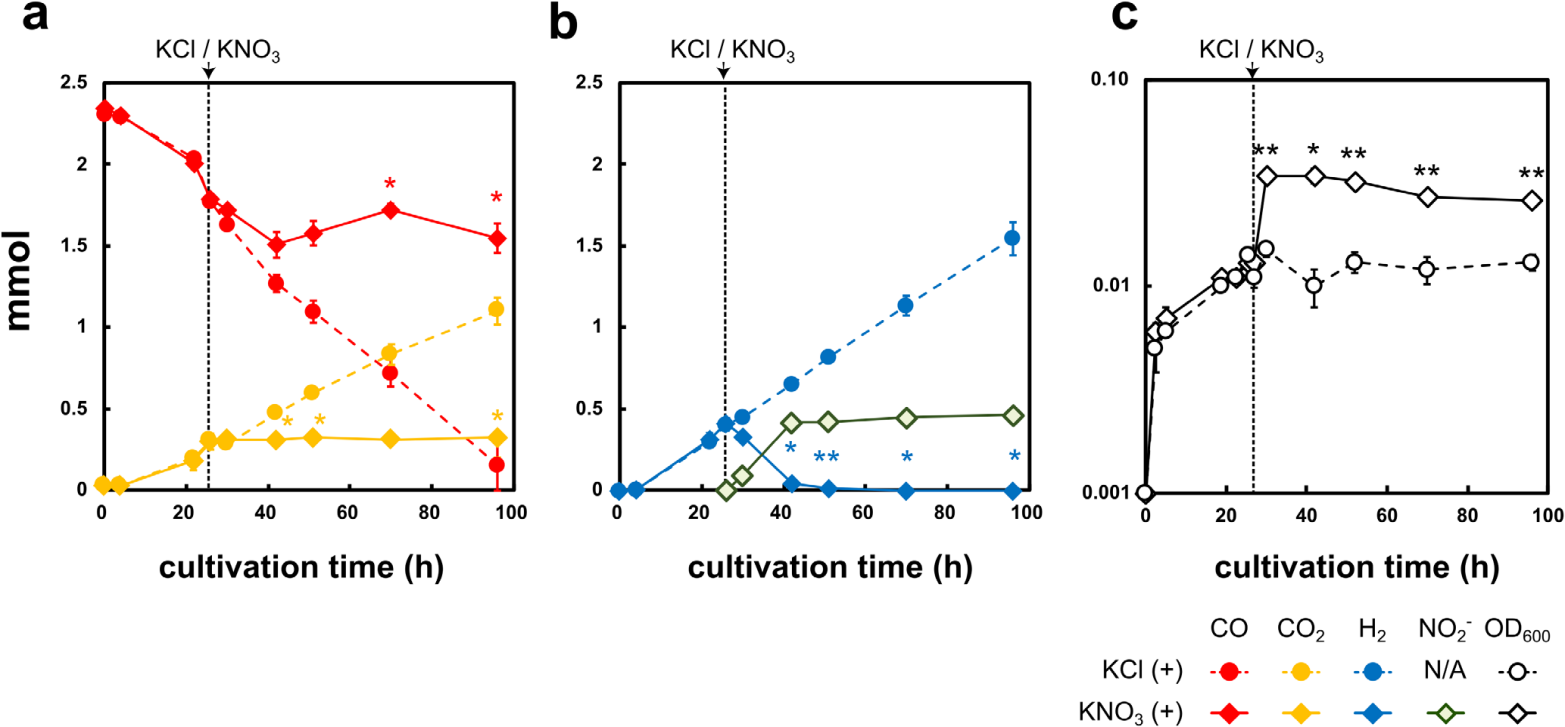
Growth of *P. thermoglucosidasius* wild‐type strain with and without addition of nitrate. The wild‐type strain was cultivated under 25% CO and 75% N_2_ atmosphere in a basal medium supplemented with 0.01% yeast extract. At 25.5 h post‐cultivation, 50 mM KNO_3_ (diamond, dotted line) or KCl (round, solid line) was added to the cultures, as indicated by the black vertical dotted lines. The total amounts of CO (red), CO_2_ (yellow) (a), H_2_ (blue), and NO_2_^−^ (light green with green lines) (b) are shown in the figure. Growth was monitored by measuring OD_600_ values (black lines) (c). The experiment was performed in triplicate. The error bars represent the standard error of the mean. Statistical analysis was performed using unpaired Welch's *t* test, and multiplicity was adjusted using Holm–Bonferroni method (*p < 0.05; **p < 0.01).

Next, we evaluated the effects of nitrate on the *P*. *thermoglucosidasius* cells that showed hydrogenogenic CO oxidation in a medium containing 0.01% yeast extract (Figure 2). After confirming hydrogenogenic CO oxidation and CO‐dependent growth at 22.5 h, 5.0 mmol (50 mM) KNO_3_ and KCl were added to the medium. During hydrogenogenic CO oxidation, the strain grew with a mean doubling time of 21.5 h, consuming CO and producing H_2_ in all tested bottles (Figure 2a–c), which is consistent with the findings of a previous study (Adachi et al. 2020). However, inoculation with KNO_3_ at 25.5 h post‐cultivation resulted in the cessation of CO consumption and the corresponding CO_2_ production (Figure 2a). In addition, 0.420 ± 0.037 mmol H_2_, produced during CO‐dependent growth, was consumed, corresponding to 0.270 ± 0.127 mmol NO_3_^−^ reduction and 0.462 ± 0.004 mmol NO_2_^−^ production on average (Figure 2b). No apparent reduction of NO_2_^−^ to NO was observed under these conditions (Figure 2b). Furthermore, during the period from 25.5 to 30 h of cultivation, when the cells utilised H_2_ and NO_3_^−^ (Figure 2b), they exhibited rapid growth with a doubling time of 3.6 h, while the OD_600_ of the KCl control remained similar to that of the KNO_3_‐inoculated cultures (Figure 2c). After depletion of H_2_, no CO consumption or growth was observed.

### 3.2 CO Oxidation, Not H_2_ Production, Was Suppressed by Nitrate

**FIGURE 3:**
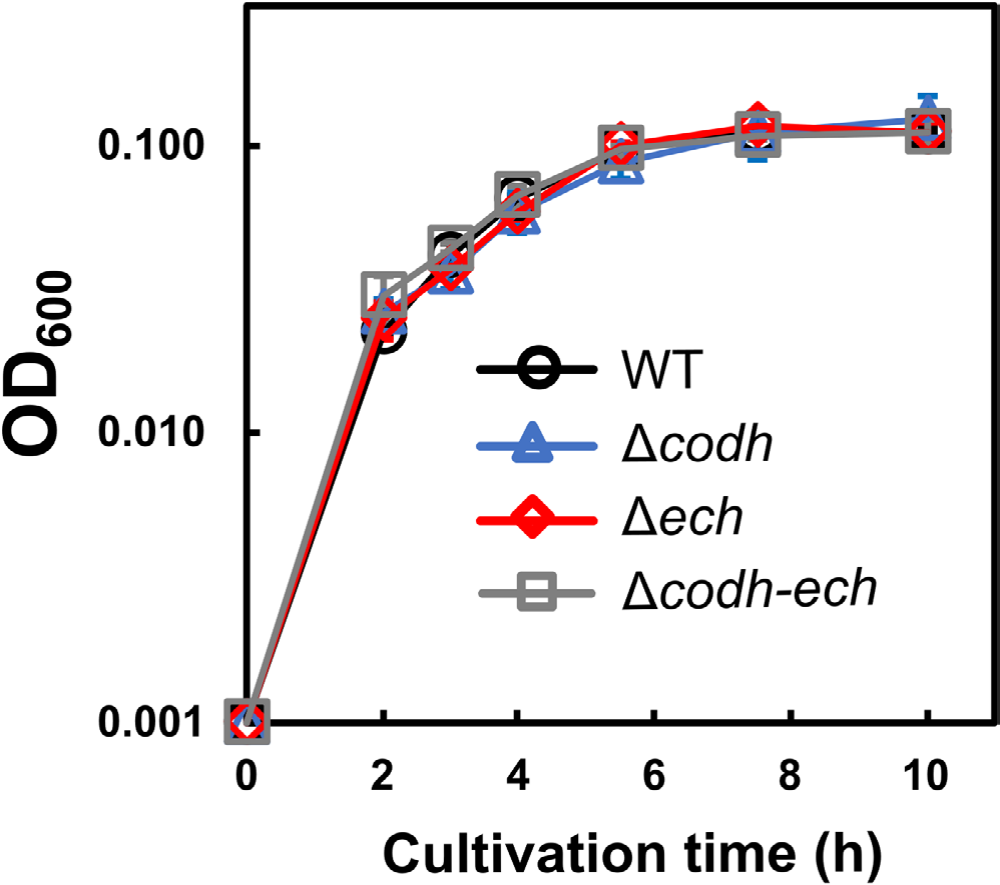
Growth of *P. thermoglucosidasius* wild‐type and *codh*–*ech*‐deleted strains during nitrate respiration. Growth using nitrate respiration in the four strains was observed in the basal medium containing 50 mM sodium pyruvate and 20 mM KNO_3_ under an N_2_ atmosphere. The experiment was performed in triplicate. The error bars represent the standard error of the mean.

To elucidate which activity (CO oxidation or H_2_ production) was being affected by nitrate in *P. thermoglucosidasius*, assays using the whole lysates of its wild‐type, Δ*codh*, Δ*ech*, and Δ*codh*–*ech* strains were performed. Mutants lacking either or both of *codh* and *ech* were previously demonstrated to have no hydrogenogenic CO‐oxidising activity (Adachi et al. 2020). Initially, growth of these strains with nitrate respiration was monitored in a culture containing 5.0 mmol (50 mM) pyruvate and 2.0 mmol (20 mM) KNO_3_ under N_2_, and no significant differences were observed in cell densities among the wild‐type and the mutant strains (Figure 3), suggesting that nitrate‐reducing ability was not altered as a result of deletion of *codh* and *ech*. To identify conditions under which Ni‐CODH/ECH is expressed in the wild‐type strain and enable comparable growth between the wild‐type and disruptant strains, cultivation of wild‐type cells was tested with 15 mM pyruvate under 25% CO with either 0% or 15% O_2_ (Figure S1). A previous study reported that, in the presence of 50% CO and 50% air, *P. thermoglucosidasius* initially grows aerobically on organic carbon sources, using these as electron donors and O_2_ as an electron acceptor; upon O_2_ depletion, hydrogenogenic CO oxidation was activated (Mohr, Aliyu, Küchlin, Polliack, et al. 2018). In this study, supplementation with 15% O_2_ supported a 2.3‐fold increase in OD_600_ (0.085 ± 0.007 vs. 0.195 ± 0.034) and a 2.9‐fold higher H_2_ production rate (21.8 ± 12.9 vs. 63.2 ± 7.39 μmol/h; calculated between 31 and 70 h) (Figure S1). Wild‐type and disruptant strains were further cultivated in 25% CO and 75% air with 20 mM glucose, resulting in comparable growth across all strains (OD_600_: wild‐type, 0.480 ± 0.008; Δ*codh*, 0.445 ± 0.004; Δ*ech*, 0.450 ± 0.033; Δ*codh*–*ech*, 0.460 ± 0.008) and hydrogenogenic CO oxidation was detected at 24 h in the wild‐type with H_2_ production rate of 58.4 ± 8.6 μmol/h (calculated between 24 and 42.5 h) (Figure S2). Given that O_2_ supplementation resulted in higher cell density, increased CO‐dependent H_2_ production activity and stable hydrogenogenic CO oxidation, the condition with both CO and air was used for subsequent assays.

**FIGURE 4:**
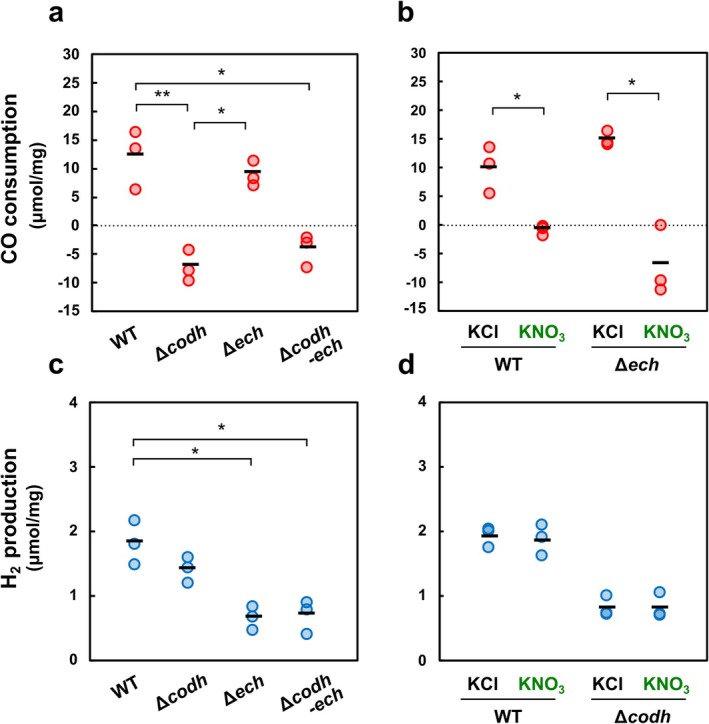
CO oxidation and H_2_ production in the whole‐cell lysates. CO‐oxidising and H_2_‐producing activities were measured using whole‐cell lysates of the wild‐type (WT), Δ*codh*, Δ*ech*, and Δ*codh*–*ech* strains. CO oxidation was measured using CO as an electron donor and MV_ox_ as an electron acceptor in the four strains (a), and in the presence of 10 μM KNO_3_ or KCl (control) (b). H_2_‐producing activity was measured using MV_red_ as an electron donor and proton as an electron acceptor in the four strains (c), and in the presence of 10 μM KNO_3_ or KCl (d). All assays were performed in triplicate. The black bars represent the mean. The statistical analysis was performed using the Tukey test for multiple comparison (a, c), and the unpaired Welch's *t* test (b, d) (*p < 0.05; **p < 0.01).

The CO‐oxidising activity was assessed using CO as an electron donor and MV_ox_ as an electron acceptor. Cultivated cells were harvested at 45 h, when the wild‐type strain was consuming CO, with mean OD_600_ values of the wild‐type and *codh*–*ech* gene‐deleted strains ranging from 0.49 to 0.75. In the lysates of the wild‐type strain, CO consumption was observed until 48 h of incubation, with nearly half of CO consumed at 24 h (data not shown). The amount of CO consumption was calculated based on the CO concentration measured at the starting and 24 h of the assays. The mean CO consumption in the lysates of the wild‐type and Δ*ech* strains was 12.1 ± 3.0 and 9.1 ± 1.2 μmol/mg (mean ± standard error), respectively. In contrast, the mean CO consumption was negative in the lysates of the Δ*codh* and Δ*codh*–*ech* strains (Δ*codh*, −7.1 ± 1.6 μmol/mg: Δ*codh*–*ech*, −4.1 ± 1.6 μmol/mg) (Figure 4a), possibly because of measurement error or CO production in the lysates. The effect of nitrate on the CO consumption was then measured in the lysates of the wild‐type and Δ*ech* strains. While culture experiments were performed with 10–50 mM of KNO_3_, suppression of CO consumption was observed at lower concentrations (10 μM) in the lysates of the wild‐type (data not shown). The lysates of the wild‐type and Δ*ech* strains showed significantly reduced consumption of CO in the absence of 50 nmol (10 μM) NO_3_^−^ (wild type, 9.9 ± 2.3 μmol/mg: Δ*ech*, 14.9 ± 0.7 μmol/mg) in comparison to in the presence of NO_3_^−^ (wild type, −0.9 ± 0.5 μmol/mg: Δ*ech*, −6.9 ± 3.5 μmol/mg) (*p* < 0.05; the unpaired Welch's *t‐*test) (Figure 4b).

H_2_ production was similarly examined using MV_red_ as the electron donor and a proton as the electron acceptor. The amount of H_2_ production was higher in the wild‐type and Δ*codh* strains (1.8 ± 0.2 and 1.4 ± 0.1 μmol/mg, respectively) than in the Δ*ech* and Δ*codh*–*ech* strains (0.7 ± 0.1 and 0.7 ± 0.1 μmol/mg, respectively), suggesting that part of H_2_ is produced by ECH (Figure 4c). H_2_ production in the lysates of ECH‐lacking strains was probably due to the reverse reaction of the two uptake hydrogenases (groups 1d/2a) present in *P. thermoglucosidasius* (Mohr, Aliyu, Küchlin, Zwick, et al. 2018), which was induced under a high concentration of MV_red_, since CO‐dependent H_2_ production was completely abolished in these strains in culture experiments (Adachi et al. 2020). No significant differences were observed in the production of H_2_ in the absence of NO_3_^−^ (wild type, 1.9 ± 0.1: Δ*codh*, 0.8 ± 0.1 μmol/mg, respectively) and presence of NO_3_^−^ (wild type, 1.9 ± 0.1: Δ*codh*, 0.8 ± 0.1 μmol/mg, respectively) (Figure 4d).

### 3.3 Comparison of Species Affected or Unaffected by Nitrate

To gain insights into species in which nitrate suppresses CO oxidation or acetate production via the WLP and those unaffected by nitrate, 13 species were compared based on our analysis and previous physiological studies (Table 1, Figure S3). *P. thermoglucosidasius* and four nitrate‐sensitive species commonly possessed clade F Ni‐CODHs, while the eight nitrate‐insensitive species lacked clade F Ni‐CODHs. No consistent patterns were observed regarding the types of nitrate reductases, the end products of nitrate reduction, or the phylogenies of the Ni‐CODH‐containing species.

## 4 Discussion

In the present study, we confirmed that nitrate suppresses hydrogenogenic CO oxidation in *P. thermoglucosidasius*. Upon the addition of nitrate to the medium, this bacterium shifted its detectable respiration from hydrogenogenic CO oxidation to H_2_‐oxidising nitrate reduction. When nitrate was added after hydrogenogenic CO oxidation commenced, electrons from H_2_, generated during hydrogenogenic CO oxidation, were transferred to nitrate. Assays using whole‐cell lysates clearly demonstrated that CO oxidation, but not H_2_ production, was suppressed by nitrate or its derivatives generated in the cell lysates. These results suggest that CO oxidation in *P. thermoglucosidasius* is coupled with proton reduction and not with nitrate reduction. These results differ from observations in *Parageobacillus* sp. G301, which couples CO oxidation to nitrate reduction (Imaura et al. 2023). Since *Parageobacillus* sp. G301 contains both Mo‐CODH and Ni‐CODH, it is possible that Mo‐CODH is necessary for the CO‐dependent nitrate reducing ability in this genus.

Hydrogenogenic CO oxidation did not begin in *P. thermoglucosidasius* as long as nitrate or nitrite remained unconsumed. Our enzyme assays demonstrated clear suppression of CO oxidation even at nitrate concentrations as low as 10 μM (Figure 4), which is lower than concentrations observed in natural ecosystems, such as forest soils (32–371 μM) (Kohlpaintner et al. 2009), deep‐sea sediments (22 μM) (Bender et al. 1977), ocean water columns (22–26 μM) (Tracey et al. 2023), and acidic hot spring muds (252 μM) (Reigstad et al. 2008). Therefore, hydrogenogenic CO oxidation by this bacterium would be limited to anaerobic environments where nitrate concentrations are low, as seen in oligotrophic marine surface waters (< 1 μM) and deep oligotrophic zones (< 22 μM) (Lewis et al. 1986), as well as strongly denitrifying soils (< 1 μM) (Knowles 1982), if the bacterium is active in these environments. Ni‐CODH/ECH‐mediated hydrogenogenic CO oxidation likely serves as a supplemental energy‐generating mechanism in these nitrate‐depleted, oligotrophic habitats, similar to the observations in aerobic CO oxidizers containing Mo‐CODH (King and Weber 2007; Greening and Grinter 2022; Fantom et al. 2025). From these observations, we conclude that Ni‐CODH/ECH is not a CO detoxification module as proposed in sulfate reducers (Parshina et al. 2010), but rather serves solely as an auxiliary energy‐obtaining module in *P. thermoglucosidasius*. The broad distribution of *P. thermoglucosidasius* may be partially supported by this CO‐oxidising system.

To the best of our knowledge, the effect of nitrate on CO utilisation has been reported in 13 prokaryotes (Table 1). In this study, we found that five CO‐utilising bacteria affected by nitrate commonly possess Ni‐CODHs classified into clade F, but not clades A or E (Table 1, Figure S3), suggesting that nitrate may affect specific clades of Ni‐CODH. However, to elucidate the mechanism underlying nitrate‐dependent suppression of CO oxidation, further experiments, such as those using purified enzymes, would be necessary.

## 5 Conclusions

This study revealed that nitrate suppresses CO oxidation in *P. thermoglucosidasius* while enhancing H_2_‐oxidising nitrate reduction and promoting rapid cell growth. Although coupling of CO oxidation and nitrate reduction theoretically enables more efficient energy generation, *P. thermoglucosidasius* did not utilise CO as a prioritised respiratory substrate. Rather, hydrogenogenic CO oxidation functions as a supplementary energy‐generating mechanism, utilised only in the absence of alternative electron acceptors in this bacterium. This study also contributes to understanding the varied relationship between nitrate and CO oxidation. Differences in nitrate effects may be associated with Ni‐CODH, but further biochemical analyses are needed to elucidate the mechanisms of nitrate‐dependent suppression of CO oxidation in certain prokaryotes.

## Author Contributions

**Yuka Adachi Katayama:** conceptualization, investigation, visualization, writing – original draft, writing – review and editing, methodology. **Yoshinari Imaura:** methodology, investigation. **Masao Inoue:** conceptualization, methodology, investigation, writing – review and editing. **Shunsuke Okamoto:** investigation. **Yoshihiko Sako:** supervision, funding acquisition, writing – review and editing. **Ryoma Kamikawa:** conceptualization, supervision, funding acquisition, methodology, writing – review and editing. **Takashi Yoshida:** conceptualization, supervision, funding acquisition, methodology, writing – review and editing.

## Conflicts of Interest

The authors declare no conflicts of interest.

## Supporting information


**Data S1.** Supporting Information.

## Data Availability

Data generated and analyzed during this study are available from the corresponding author on reasonable request.
