# Supplementary material for: Effects of Nitrate on Hydrogenogenic Carbon Monoxide Oxidation in Parageobacillus thermoglucosidasius
Source: Environ Microbiol Rep. 2025 Jun 13;17(3):e70133. doi: 10.1111/1758-2229.70133 (PMC12165953; doi:10.1111/1758-2229.70133)
Supplement: Supplementary file 1 — Data S1. Supporting Information. [file EMI4-17-e70133-s001.docx]

**Effects of nitrate on hydrogenogenic carbon monoxide oxidation**

**in *Parageobacillus thermoglucosidasius***

**Supplementary figures**

Yuka Adachi Katayama, Yoshinari Imaura, Masao Inoue, Shunsuke Okamoto,

Yoshihiko Sako, Ryoma Kamikawa, Takashi Yoshida


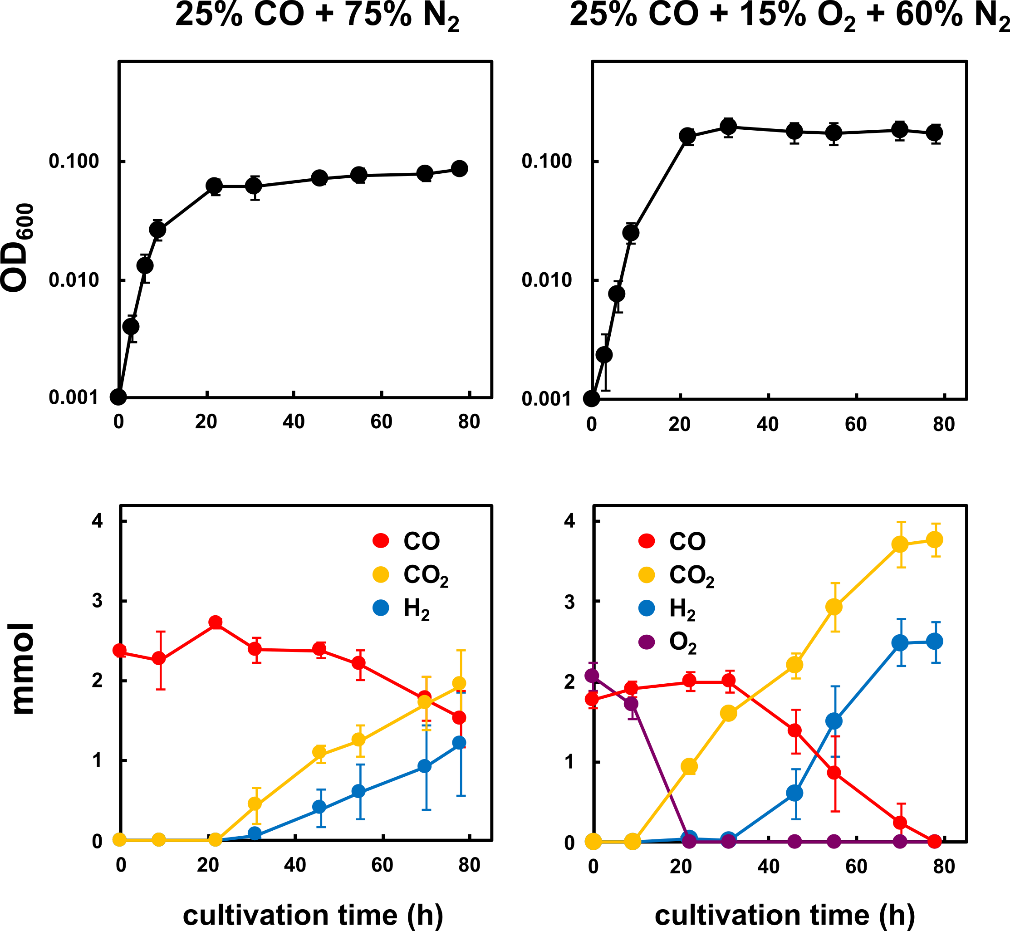


**Fig. S1.** Cultivation of *P. thermoglucosidasius* wild-type strain in the basal medium containing 15 mM sodium pyruvate. The graphs show OD_600_ values (black) and the total amounts of CO (red), CO_2_ (yellow), H_2_ (blue), O_2_ (purple) in both the headspace and liquid phase. The error bars represent the standard error of the mean.


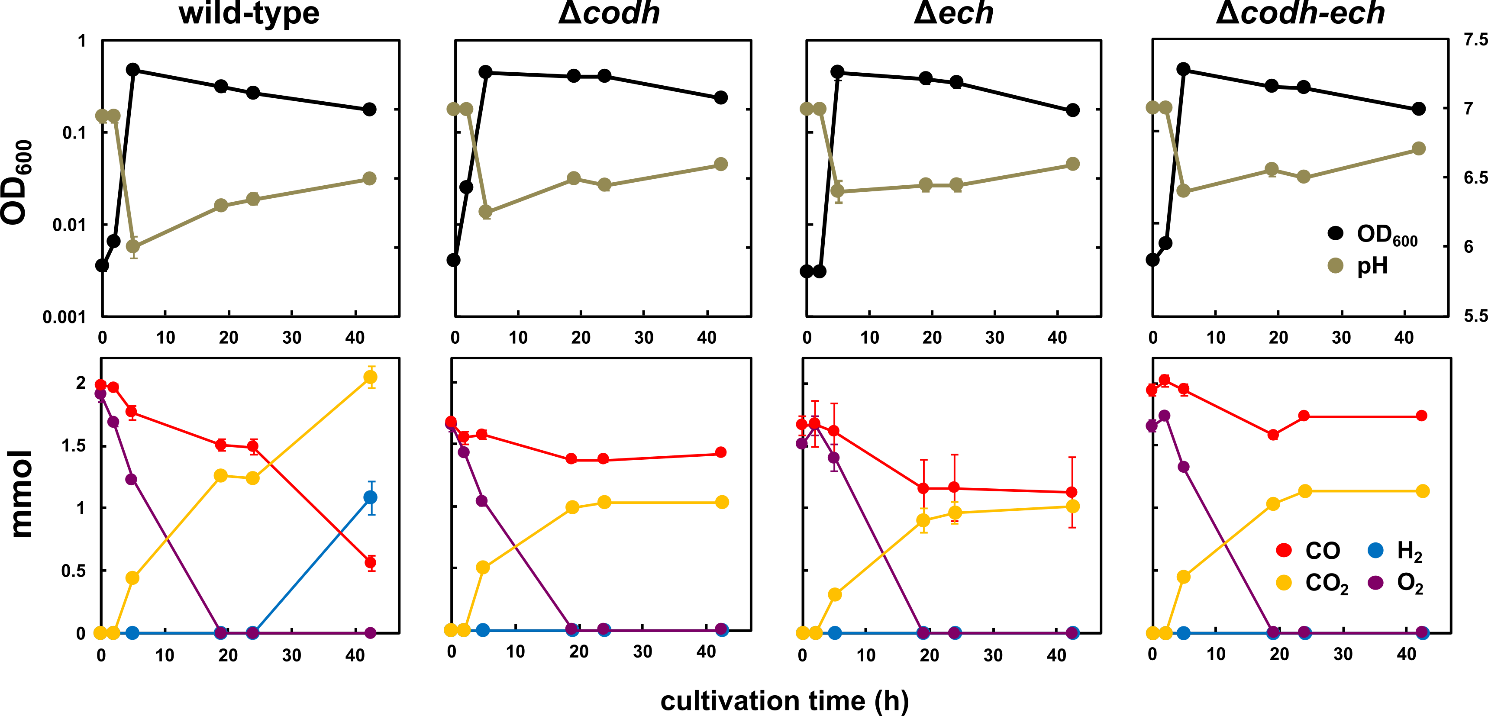


**Fig. S2.** Cultivation of *P. thermoglucosidasius* wild-type and gene disruptants under 25% CO + 75% air atmosphere. The strains were cultured in the basal medium containing 20 mM glucose and 0.1% yeast extract. The graphs show OD_600_ (black), pH (khaki), and the total amounts of CO (red), CO_2_ (yellow), H_2_ (blue), O_2_ (purple) in both the headspace and liquid phase. The error bars represent the standard error of the mean.


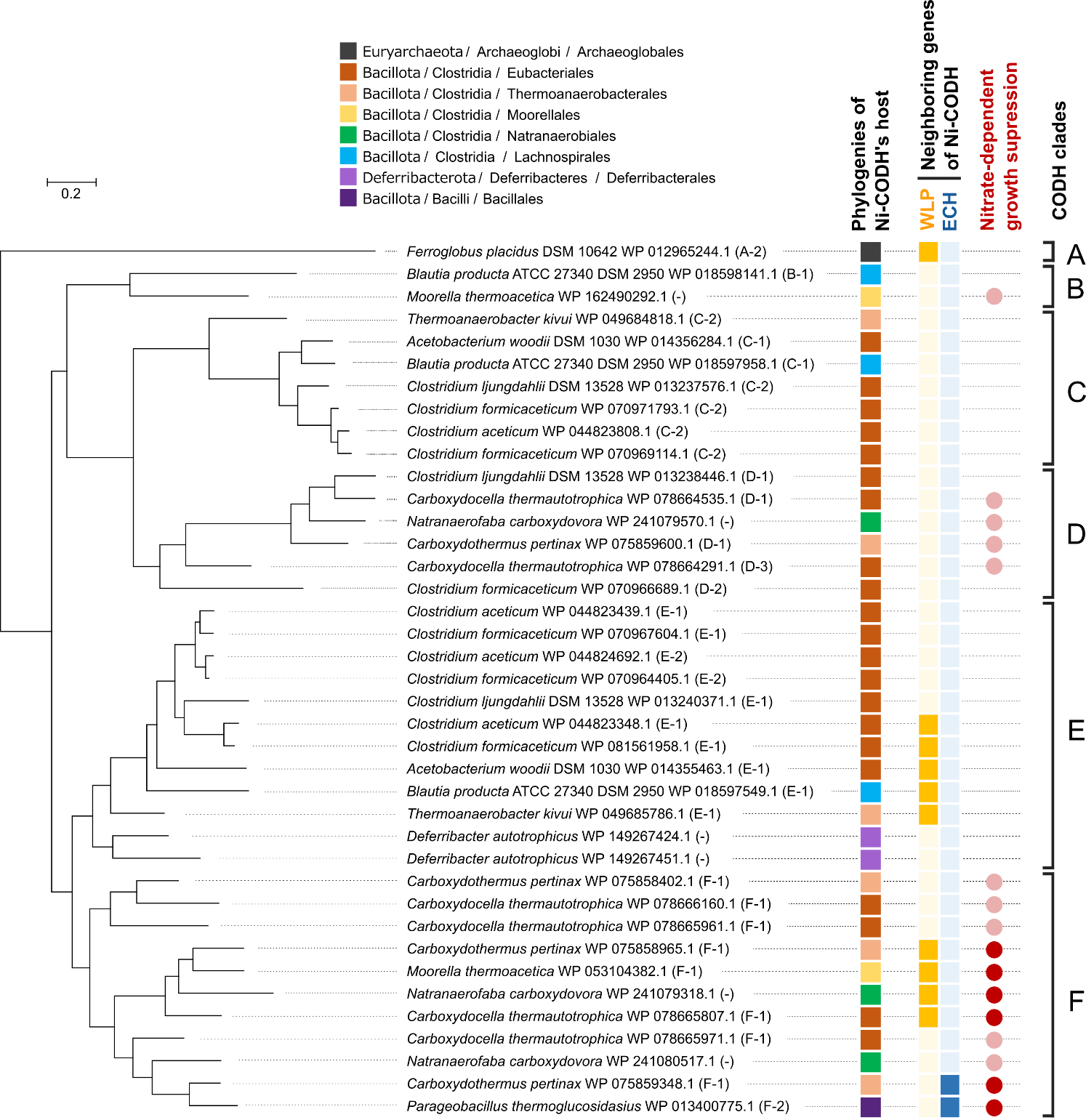


**Fig. S3.** Phylogenetic tree of 39 Ni-CODHs (CooS/CdhA) from bacteria and archaea for which nitrate-related effects have been clearly reported in previous studies. Terminal nodes indicate the name of the host organism, the RefSeq protein accession number, and previously assigned clades (Inoue *et al*., 2019), if available. The columns to the right show the phylogeny of the Ni-CODH-containing host, the presence of neighboring genes (acetyl-CoA synthase, ACS; energy-converting hydrogenase, ECH), and nitrate-dependent growth suppression. Red dots indicate Ni-CODHs that are located adjacent to either ACS or ECH in organisms where nitrate-dependent suppression has been observed. Pink dots represent others. For further details, see Table 1.
